# From toxicogenomics to predictive toxicology and exposomics: defining the next decade of gene–environment research

**DOI:** 10.3389/fgene.2026.1903791

**Published:** 2026-07-21

**Authors:** Douglas M. Ruden

**Affiliations:** Department of Obstetrics and Gynecology, Institute of Environmental Health Sciences, Charles S. Mott Center for Human Growth and Development, Wayne State University School of Medicine, Detroit, MI, United States

**Keywords:** artificial intelligence, exposome, exposomics, gene–environment interactions, longitudinal cohorts, multi-omics, precision health, precision prevention

## Abstract

Over the past 15 years, genetics has undergone a profound transformation driven by advances in next-generation sequencing, multi-omics technologies, single-cell analysis, genome editing, and artificial intelligence. Within this broader evolution, the field of toxicogenomics has expanded from studying genomic responses to environmental stressors toward a more comprehensive framework that integrates lifelong environmental exposures with genetic susceptibility. This shift is reflected in the evolution of Frontiers in Toxicogenomics into Frontiers in Predictive Toxicology and Exposomics. Modern exposomics seeks to characterize the totality of endogenous and exogenous exposures across the life course, while predictive toxicology aims to transform exposure and molecular data into actionable forecasts of disease risk and health outcomes. Emerging longitudinal cohorts, wearable sensors, high-throughput functional genomics, and machine-learning approaches are enabling unprecedented insights into dynamic gene–environment interactions. For example, large longitudinal cohorts such as the Environmental Influences on Child Health Outcomes (ECHO) program now integrate environmental, genomic, and developmental data across the life course. However, major challenges remain, including exposure measurement, data harmonization, causal inference, reproducibility, and translation into clinical and regulatory practice. Here, I discuss the scientific advances that have enabled this transition, identify the key barriers that continue to limit progress, and propose priorities for the next decade. I argue that the integration of genomics, exposomics, functional validation, and predictive analytics will redefine environmental health research and accelerate the emergence of precision prevention as a major goal of twenty-first century genetics.

## 1 Introduction: fifteen years of change

The past 15 years have witnessed a remarkable transformation in genetics. Advances in next-generation sequencing (Satam et al., 2023), functional genomics (Kim et al., 2024; Ruden, 2025), single-cell technologies (Lim et al., 2024), genome editing (Liu et al., 2025), artificial intelligence (AI) (O’Connor and McVeigh, 2025), and multi-omics integration (Alemu et al., 2025) have dramatically expanded our ability to characterize biological systems and their responses to environmental influences. As Frontiers in Genetics celebrates its 15th anniversary, it is an appropriate moment to reflect on how these advances have reshaped the field of toxicogenomics and to consider the scientific priorities that will define the next decade of gene–environment research.

When Frontiers in Genetics was launched in 2010, toxicogenomics was still a relatively young discipline. Building on the completion of the Human Genome Project and the emergence of high-throughput gene expression profiling (Taylor et al., 2024), the field focused primarily on understanding how environmental toxicants alter gene expression patterns (Singh et al., 2023). Microarrays and, later, RNA sequencing enabled investigators to identify transcriptomic signatures associated with chemical exposures, environmental stressors, and disease susceptibility (Antoszewski et al., 2026). The central question was straightforward: How do genes respond to exposure? The goal was to develop molecular biomarkers that could improve hazard identification, risk assessment, and mechanistic understanding of toxicological responses (Antoszewski et al., 2026).

Although these efforts generated important insights, they largely treated exposures as discrete events and often examined individual chemicals in isolation (Cory-Slechta et al., 2025). Human populations, however, are exposed not to single agents but to complex mixtures of environmental, occupational, dietary, behavioral, social, and endogenous factors that vary continuously throughout life (Vermeulen et al., 2020). At the same time, technological advances revealed that biological responses to environmental stressors extend far beyond changes in messenger RNA abundance. Epigenetic modifications, chromatin architecture, non-coding RNAs, proteomic alterations, metabolic pathways, and microbiome dynamics all contribute to the molecular consequences of exposure and influence disease risk (Turner, 2009; Faraji and Metz, 2026). These limitations highlighted the need for a more comprehensive framework capable of capturing the cumulative, dynamic, and interacting exposures that individuals experience throughout life, thereby motivating the emergence of exposomics.

As a result, the field has undergone a conceptual shift from toxicogenomics to exposomics (Vermeulen et al., 2020). Rather than focusing exclusively on how genomes respond to individual toxicants, exposomics seeks to characterize the totality of environmental and endogenous exposures experienced across the life course and to understand how these exposures interact with genetic and epigenetic variation to influence health (Vermeulen et al., 2020). The central question has therefore evolved into a much broader challenge: How do lifelong exposures interact with genomes and epigenomes to shape health trajectories, disease susceptibility, aging, and resilience?

The new title acknowledges that modern environmental health research must integrate exposure science, genomics, epigenomics, functional validation, computational modeling, and clinical translation. The rebranding of Frontiers in Toxicogenomics as Frontiers in Predictive Toxicology and Exposomics represents more than a change in terminology; it reflects a fundamental evolution of the field. This evolution of the journal section serves both as the motivation for and the organizing framework of the present perspective. This transition was driven by the recognition that environmental health research now extends beyond classical toxicology to encompass complex mixtures of chemical and non-chemical stressors, longitudinal exposure trajectories, endogenous biological responses, and their interactions with genetic susceptibility. Since its launch, the section has published studies spanning toxicogenomics, environmental epigenetics, computational toxicology, exposomics, biomarker discovery, and precision environmental health. The term exposomics captures this broader systems-level perspective, whereas predictive toxicology emphasizes the development of quantitative models that forecast disease risk, identify susceptible populations, and guide personalized interventions.

**FIGURE 1:**
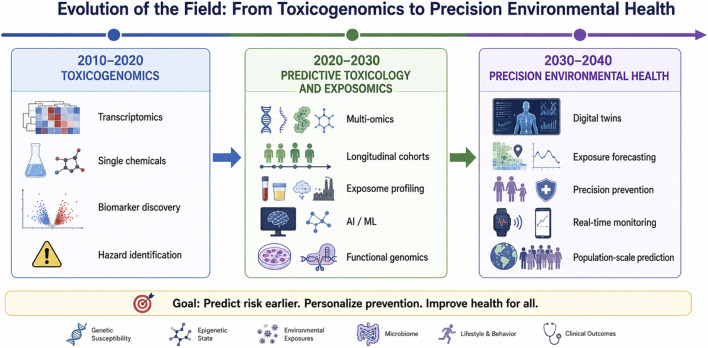
Evolution of the field from toxicogenomics to precision environmental health. Schematic overview of the conceptual and technological evolution of environmental genomics from 2010 to 2040. Left: During the toxicogenomics era (2010–2020), research focused primarily on transcriptomic responses to individual chemical exposures, emphasizing biomarker discovery and hazard identification. Middle: The central objective was to determine how genes respond to environmental stressors. The emergence of predictive toxicology and exposomics (2020–2030) expanded this framework through the integration of multi-omics technologies, longitudinal cohort studies, exposome profiling, artificial intelligence (AI), machine learning (ML), and functional genomics. These advances enabled the investigation of complex gene–environment interactions across the life course and shifted the field toward predictive modeling of exposure-associated health outcomes. Right: The projected precision environmental health era (2030–2040) will leverage digital twins, exposure forecasting systems, precision prevention strategies, real-time environmental monitoring, and population-scale predictive models to anticipate disease risk before clinical manifestation. This future vision integrates genetic susceptibility, epigenetic state, environmental exposures, microbiome composition, lifestyle factors, and clinical data within a unified predictive framework. The overarching goal of this evolution is to move from retrospective characterization of environmental effects toward proactive prediction and prevention of disease, thereby improving individual and population health outcomes through precision environmental health.

Today, emerging technologies are bringing this vision within reach. Major advances include longitudinal cohorts such as the Environmental Influences on Child Health Outcomes (ECHO) program (Knapp et al., 2023); exposure assessment technologies, including wearable sensors (Xian, 2026), and high-resolution exposome profiling (Schmitt et al., 2023); molecular profiling approaches such as single-cell multi-omics (Lim et al., 2024); functional genomics tools including CRISPR-based approaches (Kim et al., 2024; Ruden, 2025); and computational frameworks based on artificial intelligence (AI) (O’Connor and McVeigh, 2025) are generating unprecedented opportunities to understand gene–environment interactions across the lifespan. At the same time, significant challenges remain, including exposure measurement, data harmonization, causal inference, reproducibility, and translation into public health and regulatory practice. In this perspective, I examine the major advances that have driven the transition from toxicogenomics to predictive toxicology and exposomics, discuss the obstacles that continue to limit progress, and propose priorities that may help define the next era of environmental genetics (Figure 1).

## 2 What changed recently?

The transformation of toxicogenomics into predictive toxicology and exposomics did not occur because of a single breakthrough. Rather, it emerged from the convergence of multiple technological, computational, and conceptual advances that have fundamentally changed how researchers investigate the relationships between genes, environments, and health. During the past decade, four developments have been particularly influential: the rise of exposomics (Wan et al., 2025), the establishment of large longitudinal human cohorts (Manolio et al., 2020), the maturation of multi-omics and single-cell technologies (Lim et al., 2024; Wu et al., 2024), and the integration of artificial intelligence (AI) into biomedical research (O’Connor and McVeigh, 2025). Together, these advances have expanded the field from describing molecular responses to environmental exposures toward predicting health outcomes across the lifespan.

### 2.1 The rise of exposomics

The most significant conceptual advance in environmental health research has been the emergence of exposomics, which seeks to understand how cumulative and dynamic exposures influence biological systems across the life course (Wan et al., 2025). Recent advances in high-resolution mass spectrometry (Shang et al., 2025), metabolomics (Shang et al., 2025; Nafie et al., 2025), environmental sensors (Xian, 2026), geospatial monitoring (Iyer et al., 2025), and computational modeling (Iyer et al., 2025) have made comprehensive exposure profiling increasingly feasible. Emerging nanoscale detection technologies, including advanced nanopore-based sensing platforms capable of detecting individual protein molecules with high resolution, may further expand the sensitivity and specificity of molecular exposure monitoring (Yan et al., 2024; Cheng et al., 2026). These technologies now allow investigators to characterize thousands of endogenous and exogenous molecules simultaneously, providing a systems-level view of how environmental factors interact with genomic and epigenomic variation. Although these technologies have greatly expanded exposure characterization, many detected molecular features remain incompletely annotated, highlighting an important challenge that continues to limit comprehensive interpretation of untargeted exposomics datasets. As a result, exposomics has become a central framework for understanding disease etiology and gene–environment interactions in the twenty-first century. Recent studies have also extended exposomics to the characterization of environmental allergenomes, generating comprehensive catalogs of allergenic proteins that can be integrated with broader exposure assessment frameworks (Zho et al., 2026).

### 2.2 Longitudinal human cohorts and life-course exposomics

A second transformative development has been the growth of large longitudinal cohort studies that follow individuals over extended periods of time. Such studies provide an opportunity to move beyond cross-sectional snapshots and investigate how exposures accumulated across the life course influence health trajectories. However, maintaining participant engagement and minimizing attrition remain important challenges, as long-term retention directly influences data completeness and the reliability of longitudinal analyses. The Environmental Influences on Child Health Outcomes (ECHO) program is one example of this new paradigm (Knapp et al., 2023). By integrating environmental, clinical, genomic, and developmental data from tens of thousands of mother–child pairs, ECHO seeks to identify how prenatal and early-life exposures affect health outcomes throughout childhood and beyond (Knapp et al., 2023). Similar longitudinal efforts are being applied to studies of aging (Stanziano et al., 2010; Chen et al., 2026), cancer (Zhuang et al., 2026), neurodegenerative disease (Fischer et al., 2025), and chronic metabolic disorders (Aidarbekova et al., 2025; Obrador de Hevia et al., 2025; Sheikh et al., 2026). These cohorts are transforming our understanding of environmental health by enabling researchers to identify critical windows of susceptibility, characterize cumulative exposure burdens, and examine how genetic predisposition modifies exposure-associated risks. Increasingly, longitudinal exposomics studies are revealing that disease susceptibility often reflects decades of interactions between genomes and environments rather than the effects of single exposures occurring at isolated points in time.

### 2.3 Multi-omics and single-cell technologies

Another major advance has been the rapid expansion of multi-omics technologies. The integration of these diverse molecular layers provides a more comprehensive understanding of biological responses to environmental stressors. For example, exposure-induced changes in gene expression can now be linked directly to alterations in DNA methylation, histone modifications, protein signaling pathways, and metabolic networks (Axsom et al., 2021; Cheng et al., 2018). Such integrative analyses are helping to distinguish causal mechanisms from downstream biomarkers and are improving our ability to identify pathways that contribute to disease susceptibility. Recent multi-omics studies have further demonstrated the power of integrating genetic, transcriptomic, epigenomic, and proteomic information to identify key molecular mediators of disease (Ma et al., 2025). Single-cell technologies have further accelerated this transition. Rather than averaging signals across heterogeneous tissues, researchers can now investigate exposure responses at cellular resolution (Lim et al., 2024; Wu et al., 2024). Single-cell transcriptomics, epigenomics, and multi-modal profiling reveal how specific cell populations respond to environmental challenges and identify rare cellular states that may play disproportionate roles in disease pathogenesis (Lim et al., 2024; Wu et al., 2024). For example, single-cell studies have revealed rare cellular subpopulations that exhibit distinct responses to environmental stressors and toxicant exposures that would be masked in bulk tissue analyses (Shah et al., 2025). These approaches are beginning to uncover cellular mechanisms that were previously invisible in bulk analyses.

### 2.4 Artificial intelligence and predictive modeling

Perhaps the most important recent development is the emergence of AI-driven approaches capable of integrating increasingly complex biological and environmental datasets (O’Connor and McVeigh, 2025; Singh et al., 2023; Iyer et al., 2025). Modern environmental health studies routinely generate millions of measurements spanning exposures, molecular phenotypes, clinical outcomes, and demographic variables. Traditional analytical methods often struggle to identify meaningful relationships within such high-dimensional datasets. Machine learning and AI approaches offer new opportunities to address this challenge. These methods can identify complex patterns, generate predictive models, and uncover previously unrecognized interactions among genetic, environmental, and behavioral factors (O’Connor and McVeigh, 2025; Singh et al., 2023; Iyer et al., 2025; Xiang et al., 2022; Li et al., 2022; Xiang et al., 2021). Network-based approaches that integrate heterogeneous biological datasets and model information flow across molecular interaction networks have further expanded the ability of AI systems to identify disease-associated genes and predict biologically meaningful relationships (Nam et al., 2024). AI is increasingly being applied to biomarker discovery, exposure classification, disease risk prediction, toxicological screening, and precision health applications. Importantly, AI also supports the transition from descriptive to predictive science. Rather than simply documenting molecular responses after exposure has occurred, predictive models can estimate future disease risk, identify susceptible individuals, and inform targeted interventions before pathology develops (O’Connor and McVeigh, 2025; Singh et al., 2023; Iyer et al., 2025). Emerging concepts such as digital twins—computational representations of individuals that integrate genomic, exposomic, physiological, and clinical information—exposure forecasting systems, and personalized risk assessment frameworks illustrate the potential of AI to transform environmental health research (Silva and Vale, 2025). These advances have established the foundation for the next- generation of predictive toxicology and exposomics. Beyond prediction and classification, AI-driven approaches are increasingly being used to design novel chemical entities and evaluate their properties *in silico*, potentially accelerating toxicity screening and hazard assessment (Kim and Choi, 2025).

Although exposomics has broadened the scope of environmental health research, it should not be viewed as a replacement for toxicogenomics but rather as its natural extension. The two disciplines address complementary aspects of gene–environment interactions. Exposomics seeks to comprehensively characterize the external and internal exposures experienced across the life course, whereas toxicogenomics investigates the molecular, cellular, and physiological responses elicited by those exposures. Individually, each approach provides only part of the picture; together they offer a more complete framework for understanding how environmental factors influence health and disease. Integrating exposomic measurements with transcriptomic, epigenomic, proteomic, metabolomic, and functional genomic analyses enables investigators to move beyond exposure assessment or biomarker discovery toward mechanistic models that identify causal pathways, predict disease risk, and guide preventive interventions. This complementary relationship forms the foundation of predictive toxicology and supports the broader goal of precision environmental health.

## 3 What still blocks progress?

Despite remarkable technological advances, predictive toxicology and exposomics have not yet achieved their full potential. While researchers can now generate unprecedented volumes of molecular and exposure data, the ability to translate these measurements into reliable predictions of disease risk remains limited. In many respects, the challenges facing the field have shifted from data generation to data interpretation, integration, validation, and implementation. Four obstacles are particularly important: incomplete exposure measurement, difficulties distinguishing correlation from causation, data fragmentation, and barriers to clinical and regulatory translation.

### 3.1 Exposure measurement remains the weakest link

The Human Genome Project provided a stable reference sequence that transformed genetics (Taylor et al., 2024). Exposomics, by contrast, faces a far more difficult challenge because exposures are dynamic, multidimensional, and continuously changing throughout life. Unlike the genome, the exposome has no fixed reference state. Although advances in mass spectrometry, wearable sensors, remote sensing, and environmental monitoring have greatly expanded exposure assessment capabilities, current technologies still capture only a fraction of the exposures experienced by an individual. Many environmental chemicals remain poorly characterized, while non-chemical stressors such as psychosocial stress, socioeconomic factors, infectious agents, noise, heat, and lifestyle behaviors are often difficult to quantify. Furthermore, exposures rarely occur independently. Humans encounter complex mixtures whose components may interact synergistically or antagonistically, creating biological effects that cannot be predicted from individual agents alone. Temporal resolution presents an additional challenge. Exposures vary across hours, days, years, and decades, yet most studies rely on a limited number of sampling points. Important developmental windows—including prenatal development, childhood, puberty, reproductive aging, and senescence—may therefore be missed entirely. Identifying such critical windows depends on sufficiently dense longitudinal sampling, underscoring the importance of well-designed cohort studies capable of capturing exposure dynamics across key stages of development and aging. Until exposure assessment approaches become more comprehensive, longitudinal, and standardized, exposomics will continue to face limitations analogous to attempting to understand genetics using only a small fraction of the genome.

### 3.2 Correlation versus causation

The rapid growth of multi-omics datasets has enabled the discovery of thousands of associations linking environmental exposures to molecular signatures and disease outcomes. However, identifying associations is not equivalent to understanding biological mechanisms (He et al., 2020). Many reported biomarkers may represent downstream consequences rather than causal drivers of disease. For example, genetic polymorphisms may be associated with susceptibility to infectious diseases such as tuberculosis, yet determining whether these relationships reflect causal mechanisms or linkage to other functional variants often requires additional experimental validation (Aravindan, 2019; Chen et al., 2019). Likewise, environmental exposures frequently correlate with one another, making it difficult to determine which factors are biologically relevant. Large datasets can produce statistically significant relationships that may lack mechanistic significance or reproducibility. The field increasingly recognizes the need to move beyond association studies toward causal inference and functional validation.

Several complementary causal inference approaches have recently emerged to strengthen evidence from observational studies. Mendelian randomization uses inherited genetic variants as instrumental variables to estimate causal effects when appropriate genetic instruments are available (Sanderson et al., 2022). Target trial emulation applies principles of randomized clinical trial design to longitudinal observational datasets, reducing bias when randomized studies are impractical (Matthews et al., 2022). Causal mediation analysis can distinguish direct effects of environmental exposures from those operating through intermediate molecular pathways (McGrath et al., 2026), whereas causal machine learning approaches integrate high-dimensional datasets to identify complex causal relationships and heterogeneous treatment or exposure effects (Rudolph et al., 2019). Together, these methods provide complementary frameworks for prioritizing hypotheses for experimental validation and translating observational associations into mechanistic understanding.

Advances in CRISPR-based genome editing (Kim et al., 2024; Ruden, 2025), high-throughput perturbation screens (Oberlin et al., 2026), organoid systems (Han et al., 2026), and single-cell functional genomics (Filipovic et al., 2024) provide powerful tools for testing mechanistic hypotheses generated by exposomics analyses. The integration of observational human studies with experimental validation will be essential for transforming predictive toxicology into a truly mechanistic science. When direct experimental validation is not immediately feasible, convergent evidence from multiple complementary approaches—including longitudinal studies, Mendelian randomization, natural experiments, and independent cohort replication—may provide a practical framework for strengthening causal inference while mechanistic studies are being developed (Zuber et al., 2022). Future success will depend not only on identifying biomarkers but also on understanding how exposures alter biological pathways and how those alterations contribute to disease development.

### 3.3 Data fragmentation and lack of standardization

A paradox of modern biology is that researchers possess more data than ever before yet often struggle to combine datasets generated by different laboratories, technologies, or study designs. This problem is particularly acute in exposomics because the field integrates information from diverse disciplines, including environmental science, toxicology, genomics, epidemiology, bioinformatics, and clinical medicine. Differences in analytical platforms, sample processing methods, metadata collection, exposure definitions, and statistical approaches frequently limit cross-study comparisons. Even early analytical decisions involving quality filtering, normalization, and batch correction can substantially influence downstream results and contribute to inconsistencies among studies (He et al., 2020; Yu et al., 2024). Even when datasets are publicly available, inconsistent annotation and incomplete metadata can hinder reuse and reproducibility. As a result, many findings remain difficult to replicate across populations and laboratories. The challenge extends beyond technical standardization. Harmonized ontologies, common data elements, reference materials, and quality-control frameworks remain underdeveloped relative to those available in genomics. The success of genomics was driven in part by community-wide standards and shared databases (Kaye et al., 2009). Encouragingly, initiatives promoting FAIR (Findable, Accessible, Interoperable, and Reusable) data practices and harmonized metadata standards are already helping to address these challenges, although broader adoption remains necessary (de Groot et al., 2024). Exposomics will require a similar commitment to data harmonization and open science if it is to realize its full potential. Artificial intelligence further highlights the importance of standardized data. Machine-learning models are only as reliable as the datasets used to train them. Without robust, diverse, and interoperable datasets, predictive algorithms risk generating biased or non-generalizable conclusions.

### 3.4 Translation to public health and regulatory decision-making

Perhaps the greatest challenge facing predictive toxicology and exposomics is translating scientific discoveries into meaningful improvements in human health. While researchers increasingly identify molecular signatures associated with exposure and disease, relatively few have been incorporated into clinical practice, public health programs, or regulatory frameworks. Several barriers contribute to this gap. First, predictive biomarkers often require extensive validation across diverse populations before they can be used confidently in clinical or regulatory settings (Ou et al., 2021). Second, regulatory agencies traditionally rely on established toxicological testing paradigms that may not readily incorporate complex multi-omics or AI-derived endpoints (Kumbhar et al., 2026). Third, health disparities and population diversity complicate efforts to develop universally applicable predictive models (Gupta et al., 2025; Triwiyanto and Luthfiyah, 2026). Biomarkers or risk algorithms developed in one population may perform poorly in others if underlying genetic, environmental, or socioeconomic contexts differ (Hegarty et al., 2025). Addressing this challenge is particularly important as precision health approaches expand, since inadequate representation of diverse populations may inadvertently widen existing health disparities and reduce the generalizability of predictive models. Ethical considerations further complicate implementation. Increasingly detailed exposure and genomic datasets raise concerns regarding privacy, data ownership, informed consent, and equitable access to precision-health technologies. Emerging governance approaches, including federated data-access models that allow analyses to be performed across distributed datasets without transferring sensitive individual-level information, may help balance scientific collaboration with privacy protection (Casaletto et al., 2023). Addressing these issues will require collaboration among scientists, clinicians, policymakers, ethicists, and affected communities.

The ultimate measure of success for predictive toxicology and exposomics will not be the number of biomarkers discovered or datasets generated. Rather, it will be the ability to improve risk assessment, identify vulnerable populations before disease develops, guide preventive interventions, and reduce environmentally driven health disparities. Achieving these goals will require overcoming the scientific and societal barriers that currently limit translation. Collectively, these challenges illustrate that the future of predictive toxicology and exposomics depends not merely on generating more data but on generating better data, integrating information across disciplines, validating causal mechanisms, and developing frameworks that allow discoveries to influence public health and clinical decision-making. Addressing these barriers should therefore be a central priority for the coming decade.

## 4 What standards are needed for the next decade?

The extraordinary advances described above have created unprecedented opportunities for predictive toxicology and exposomics. However, technological innovation alone will not guarantee scientific progress. The history of genomics demonstrates that transformative advances require not only new tools but also shared standards, reference datasets, quality-control frameworks, and community-wide best practices. The Human Genome Project succeeded because researchers adopted common protocols, standardized data formats, and openly accessible resources that enabled reproducibility and collaboration across institutions and countries (Taylor et al., 2024). Predictive toxicology and exposomics now face a similar inflection point. As datasets become increasingly large and complex, the absence of harmonized standards threatens to limit interoperability, reproducibility, and translation. To realize the promise of precision environmental health, the field must establish a robust framework for exposure measurement, longitudinal cohort integration, functional validation, and ethical governance.

### 4.1 Standardizing the exposome

One of the highest priorities for the coming decade is the development of standardized approaches for measuring and describing environmental exposures. While genomic data benefit from universally accepted reference genomes and annotation systems, exposomics studies often employ different analytical platforms, exposure definitions, sampling strategies, and reporting conventions. These inconsistencies make it difficult to compare results across studies and populations. Future progress will require the establishment of common exposure ontologies, standardized metadata requirements, and reference materials that facilitate cross-study harmonization. Researchers should adopt FAIR (Findable, Accessible, Interoperable, and Reusable) data principles whenever possible and develop community standards for reporting environmental, occupational, dietary, psychosocial, and endogenous exposures (Crystal-Ornelas et al., 2022). Originally developed to promote data sharing and interoperability across scientific disciplines, including Earth and environmental sciences, FAIR principles are increasingly being adapted to address the growing complexity of biomedical and exposomic datasets (Heacock et al., 2022). Such efforts would greatly improve reproducibility and allow exposomics datasets generated by different groups to be integrated into larger analytical frameworks. Advances in wearable sensors, remote monitoring technologies, and digital health platforms further highlight the need for standardized approaches to collecting, storing, and interpreting exposure data. Without common frameworks, the increasing volume of exposure information may generate fragmentation rather than scientific insight.

### 4.2 Building benchmark longitudinal cohorts

A second priority is the establishment of benchmark longitudinal cohorts that can serve as foundational resources for environmental health research. Just as projects such as the 1000 Genomes Project (Gustafson et al., 2024; Hanchard and Choudhury, 2022; Siva, 2008), UK Biobank (Sudlow et al., 2015), and The Cancer Genome Atlas (Hong et al., 2025; McCain, 2006) transformed human genetics, large, well-characterized exposome cohorts have the potential to accelerate discovery across multiple disciplines. Unlike genomic reference datasets, however, exposome data are inherently dynamic, context-dependent, and population-specific. Consequently, benchmark exposome cohorts may require adaptive sampling strategies and flexible data architectures capable of accommodating changing environmental conditions and exposure profiles over time (Luan and Daoyu, 2025). Attention should be given to identifying critical developmental windows during which environmental exposures exert disproportionate biological effects. International collaboration and harmonized study designs will be essential for generating datasets sufficiently large and diverse to support robust predictive modeling. Programs such as ECHO illustrate how harmonized longitudinal cohorts can provide a foundation for integrating exposomics, genomics, and health outcomes across the lifespan (Knapp et al., 2023).

### 4.3 Establishing functional validation frameworks

The next decade must also place greater emphasis on functional validation. While high-dimensional datasets can identify thousands of exposure-associated biomarkers, relatively few are subjected to rigorous mechanistic testing. As a result, the field risks accumulating extensive catalogs of correlations without establishing causal relationships. Integrating observational human data with experimental validation should become a standard component of predictive toxicology research. Equally important is the development of benchmark datasets for evaluating predictive models. Independent replication cohorts, blinded validation studies, and standardized performance metrics will help ensure that proposed biomarkers and risk prediction algorithms are robust, reproducible, and clinically meaningful. As AI-driven approaches become increasingly common, rigorous validation standards will be essential for preventing overfitting and ensuring generalizability across populations.

### 4.4 Regulatory and implementation frameworks

The ultimate success of predictive toxicology and exposomics will depend on whether scientific advances can be translated into regulatory and public health practice. As multi-omics biomarkers, exposomics measurements, and AI-based prediction models become increasingly sophisticated, clear frameworks will be needed to evaluate their reliability, reproducibility, and fitness for decision-making. Such frameworks may include independent validation using external datasets, transparent reporting of model performance metrics, assessment of robustness across diverse populations, and ongoing evaluation of algorithmic bias and interpretability. Regulatory agencies, academic researchers, and industry stakeholders should work together to establish evidence-based criteria for validating new approaches and incorporating them into risk assessment workflows. Such frameworks will help ensure that innovations in predictive toxicology move beyond research settings and contribute meaningfully to environmental health protection, disease prevention, and precision public health.

## 5 Conclusions and future directions

This transformation is reflected in the evolution of Frontiers in Toxicogenomics into Frontiers in Predictive Toxicology and Exposomics. More broadly, it reflects a shift toward integrating mechanistic understanding with predictive capability, enabling environmental health research to move beyond documenting exposure effects toward anticipating disease risk and informing prevention strategies. What began as a field largely focused on measuring transcriptional responses to environmental toxicants has evolved into a far broader effort to understand how the totality of exposures experienced throughout life influences biological function, disease susceptibility, and health outcomes.

Building upon this conceptual transition, the convergence of exposomics, longitudinal cohort studies, multi-omics profiling, functional genomics, and AI-driven analytics has created an unprecedented opportunity to move from observation toward prediction in environmental health research. The convergence of exposomics, longitudinal cohort studies, multi-omics profiling, functional genomics, and AI-driven analytics has created an unprecedented opportunity to achieve this goal. For the first time, researchers can begin to construct comprehensive models that integrate environmental exposures, genetic susceptibility, molecular responses, and clinical outcomes within a unified framework. These advances are bringing the field closer to a future in which disease risk can be anticipated before pathology develops and preventive interventions can be tailored to the unique biological and environmental context of everyone.

Despite this progress, significant challenges remain. Comprehensive exposure measurement, data harmonization, mechanistic validation, reproducibility, and clinical translation continue to limit the full realization of predictive toxicology and exposomics. Addressing these challenges will require the same spirit of collaboration, standardization, and open science that enabled the success of modern genomics. Establishing shared exposure ontologies, benchmark longitudinal cohorts, interoperable databases, functional validation pipelines, and transparent ethical frameworks should therefore be viewed as central priorities for the next decade.

Looking forward, several trends are likely to define the future of the field. Real-time exposure monitoring, AI-assisted risk prediction, digital twins, precision prevention strategies, and globally integrated exposome networks have the potential to transform both biomedical research and public health practice. As these technologies mature, predictive toxicology and exposomics may become essential components of precision health, allowing environmental risks to be identified, quantified, and mitigated before disease occurs. The broader genetics community is also entering a new phase. The challenge is no longer simply generating genomic or molecular data. Rather, it is integrating diverse sources of information into mechanistic and predictive frameworks that improve human health. In this sense, predictive toxicology and exposomics exemplify a central theme of the next era of genetics: understanding how inherited biological information interacts with the environments we experience throughout life.

The central challenge for the field remains the same question introduced at the outset of this perspective: how do lifelong exposures interact with genomes and epigenomes to shape health trajectories, disease susceptibility, aging, and resilience? Ultimately, the next 15 years will determine whether predictive toxicology and exposomics can move beyond measuring the exposome to predicting its consequences. If successful, the field will help transform environmental health from a largely reactive discipline into a science of precision prevention, enabling earlier interventions, reducing environmentally driven disease, and improving health outcomes across populations worldwide.
